# Noncoding RNAs as an emerging resistance mechanism to immunotherapies in cancer: basic evidence and therapeutic implications

**DOI:** 10.3389/fimmu.2023.1268745

**Published:** 2023-09-12

**Authors:** Man Wang, Fei Yu, Peifeng Li

**Affiliations:** Institute for Translational Medicine, The Affiliated Hospital of Qingdao University, College of Medicine, Qingdao University, Qingdao, China

**Keywords:** cancer, tumor microenvironment, immunotherapy resistance, ncRNAs, tumor immunity, ncRNA-based therapies

## Abstract

The increasing knowledge in the field of oncoimmunology has led to extensive research into tumor immune landscape and a plethora of clinical immunotherapy trials in cancer patients. Immunotherapy has become a clinically beneficial alternative to traditional treatments by enhancing the power of the host immune system against cancer. However, it only works for a minority of cancers. Drug resistance continues to be a major obstacle to the success of immunotherapy in cancer. A fundamental understanding of the detailed mechanisms underlying immunotherapy resistance in cancer patients will provide new potential directions for further investigations of cancer treatment. Noncoding RNAs (ncRNAs) are tightly linked with cancer initiation and development due to their critical roles in gene expression and epigenetic modulation. The clear appreciation of the role of ncRNAs in tumor immunity has opened new frontiers in cancer research and therapy. Furthermore, ncRNAs are increasingly acknowledged as a key factor influencing immunotherapeutic treatment outcomes. Here, we review the available evidence on the roles of ncRNAs in immunotherapy resistance, with an emphasis on the associated mechanisms behind ncRNA-mediated immune resistance. The clinical implications of immune-related ncRNAs are also discussed, shedding light on the potential ncRNA-based therapies to overcome the resistance to immunotherapy.

## 1 Introduction

Immunotherapy is a type of cancer treatment that harnesses the body’s immune system to target and eliminate cancer cells. Currently, immunotherapy has become a main weapon in fighting cancer (1). Several immunotherapy approaches, such as adoptive cellular immunotherapy and immune checkpoint inhibitor (ICI), have been approved for the treatment of various cancers including melanoma, non-small cell lung cancer (NSCLC) and renal cell carcinoma (RCC) (2). These immunotherapies can achieve long-term tumor regression in subsets of patients. Despite the encouraging improvement in the field of cancer immunotherapy, a significant proportion of cancer patients do not benefit from immunotherapy and some responding patients even undergo relapse after a period of treatment, implying a role of immune resistance (3). The response rates also differ between distinct immunotherapeutic modalities and across cancer types (4). Thus, there is an urgent need to bypass cancer resistance in order to maximize the therapeutic benefits of cancer immunotherapy. Immunotherapy resistance can be generally categorized into intrinsic and acquired resistance (5). Intrinsic resistance, also known as primary resistance, represents a clinical situation where a cancer exhibits low responsiveness to immunotherapy (6). Acquired resistance represents a clinical scenario in which a cancer initially responds to immunotherapy, but later evolves multifarious mechanisms to relapse or progress (6). The mechanisms of cancer immune resistance are very intricate and involve many aspects including genes and metabolism. Particularly, alternation of antitumor immune response pathways and intracellular signaling cascades, abnormal expression of tumor antigens, disruption of antigen presentation processes, formation of an immunosuppressive microniche, functional gene mutations, changes in metabolism in the tumor microenvironment, as well as host-related factors have been associated with immune resistance in cancer (5). An in-depth exploration of immune resistance mechanisms will facilitate the discovery of new therapeutic targets and extend the scope of clinical applications of immunotherapeutic treatments.

Noncoding RNAs (ncRNAs) that account for the vast majority (~98%) of the transcribed genome are a broad and heterogeneous group of RNA molecules in terms of origin, structure, biogenesis and biological function (7, 8). NcRNAs have emerged as ubiquitous players in numerous cellular processes under both the physiological and pathological conditions (9, 10). Based on a length cut-off of 200 nucleotides (nt), ncRNAs are roughly grouped into two classes: short ncRNAs (18-200 nt) and long ncRNAs (lncRNAs, >200 nt) (11, 12). Short ncRNAs include microRNAs (miRNAs), piwi-interacting RNAs (piRNAs) and small nucleolar RNAs (snoRNAs) (13, 14). miRNAs, the most extensively studied type of short ncRNAs, have important roles as regulators of gene expression (15, 16). miRNAs directly bind to the 3’ untranslated region (3’ UTR) of target mRNAs to induce mRNA degradation or in some instances repress protein translation (17). LncRNAs can be further classified into bidirectional transcripts, enhancer RNAs (eRNAs), long intergenic ncRNAs (lincRNAs) and natural antisense transcripts (NATs) (18, 19). Accumulating evidence has demonstrated that lncRNAs act on gene regulation at the epigenetic, transcriptional, post-transcriptional and translational levels via diverse mechanisms (20, 21). Circular RNAs (circRNAs) are recently discovered as a peculiar class of lncRNAs, which form covalently closed continuous loops and are extensively present in mammalian cells (22, 23). CircRNAs can be generally divided into exonic circRNAs (ecircRNAs), circular intronic RNAs (ciRNAs), exon-intron circRNAs (EIciRNAs), and intergenic circRNAs (24–26). CircRNAs act as molecular sponges for miRNAs and proteins, modulators of alternative splicing and transcription and peptide/protein translators (27, 28).

Currently, ncRNAs have become a new research hotspot in the field of cancer immunotherapy. A growing body of evidence has manifested that miRNAs, lncRNAs and circRNAs have a role in regulating the diverse stages of tumor immunity through modulation of the equilibrium between immune activation and immunosuppression (29, 30). These ncRNAs are involved in mediating the communication between cancer cells and immune cells and represent crucial resistance mechanisms that can abrogate the effect of immunotherapy (31, 32). Therefore, ncRNAs may be prospective therapeutic targets to improve the success of immunotherapy in cancer patients. In this review, we present the current state of knowledge implicating miRNAs, lncRNAs and circRNAs in shaping cancer resistance to immunotherapy, with a particular focus on the underlying mechanisms. We also discuss their potential in developing efficacious therapeutic agents for cancer treatment.

## 2 Cancer immunotherapy

**Figure 1:**
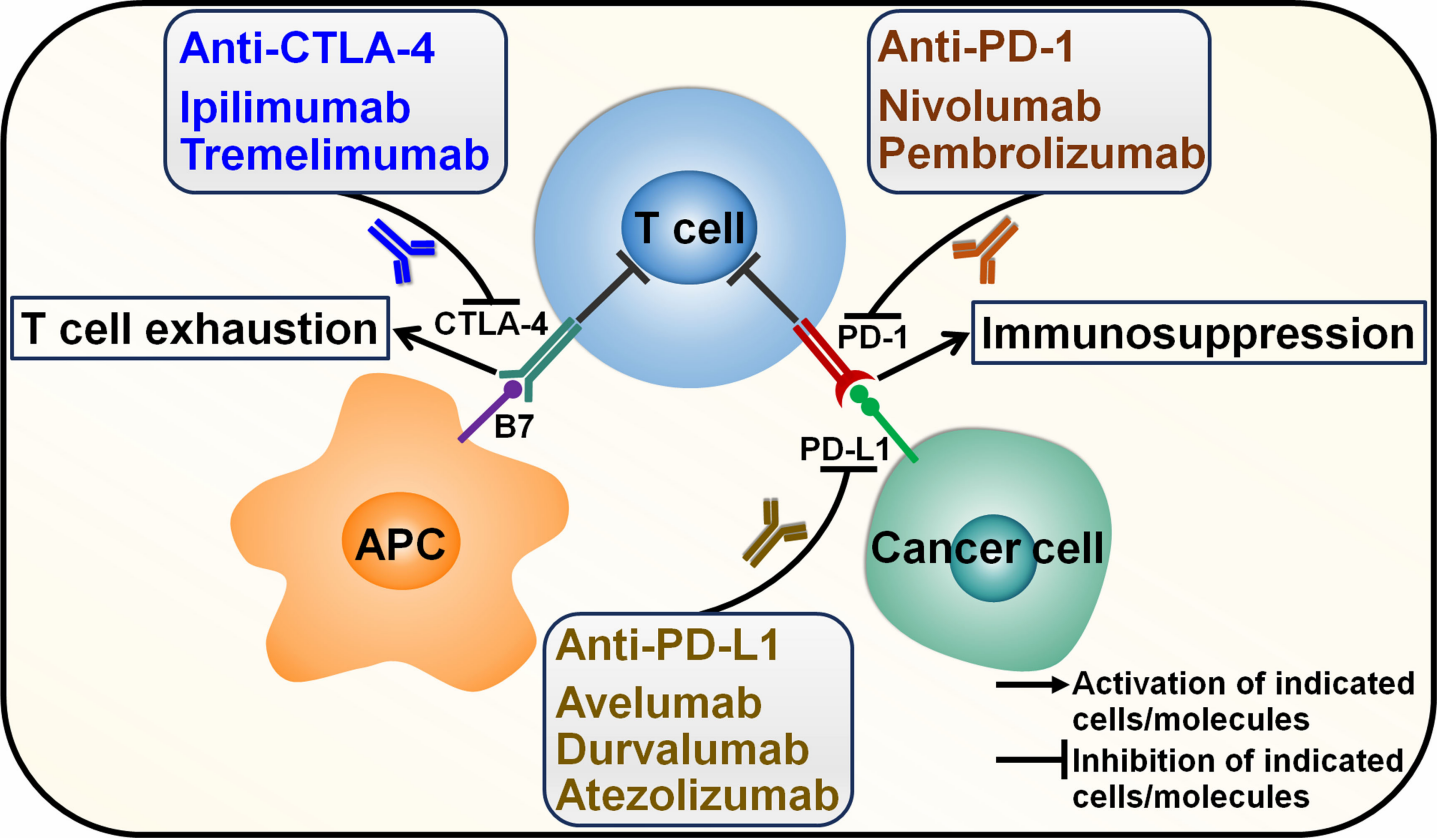
Mechanisms of action of immune checkpoint inhibitors. Cancer cells secrete cancer-associated antigens that are captured by APCs through the MHC-I molecule. APCs then activate T cells, which in turn kill cancer cells. However, cancer cells have evolved multifarious mechanisms to evade immune surveillance. Particularly, immune checkpoints that are expressed on cancer cells and cancer-specific lymphocytes can inhibit T cell activation. The most extensively studied immune checkpoints are CTLA-4, PD-L1 and PD-1. Activation of T cells requires the interaction between CD28 expressed on T cells and B7 on APC. CTLA-4 on T cells binds B7, competing with CD28, and suppresses T cell activation. PD-1 is an inhibitory receptor expressed on T cells. The interaction between PD-1 and its ligand PD-L1 induces T cell exhaustion. Immune checkpoint inhibitors (ICIs) are monoclonal antibodies that specifically target immune checkpoints. ICIs can impede the inhibitory signals and reinvigorate anticancer immune responses. Two monoclonal antibodies ipilimumab and tremelimumab have been developed to inhibit CTLA-4. Nivolumab and pembrolizumab are monoclonal antibodies that target PD-1. Monoclonal antibodies against PD-L1 include avelumab, durvalumab and atezolizumab. CTLA-4, cytotoxic T lymphocyte-associated antigen-4; APC, antigen-presenting cell; PD-1, programmed cell death protein-1; PD-L1, programmed cell death-ligand 1.

Cancer immunotherapy eliminates cancer cells by various steps in cancer-immunity cycle including antigen presentation, T cell priming and activation and immune cell-mediated cytotoxic effects (33). Tumor-specific T cells can be activated through recognition of a major histocompatibility complex (MHC)-bound cancer epitope by the T cell receptor (TCR) or the interaction between co-stimulatory receptors (e.g., CD28) on T cells and their corresponding ligands (e.g., B7 molecules) on activated antigen-presenting cells (34). Co-stimulatory (e.g., CD27 and OX40) and co-inhibitory molecules (e.g., cytotoxic T lymphocyte-associated antigen-4 (CTLA-4) and programmed cell death protein-1 (PD-1)) play a pivotal role in T cell function. The dynamic interaction between stimulatory and inhibitory signals on T cells controls the extent of immune activation to ensure tolerance to self-antigens (inhibitory), while mounting an effective adaptive immune response to exogenous antigens (stimulatory) (35). The engagement of co-inhibitory checkpoint molecules such as CTLA-4 and PD-1 by their respective counterparts can block T cell expansion and activation, culminating in the immune evasion of cancer cells (36) (**Figure 1**).

ICI therapy functions to impede immune checkpoint-mediated suppression and thus rescues the activity of tumor-specific T cells (37). The introduction and clinical implementation of T cell-directed immunomodulators blocking immune checkpoints CTLA-4, programmed cell death-ligand 1 (PD-L1) and PD-1 have rejuvenated the field of tumor immunology and revolutionized cancer treatment far beyond their impressive clinical activity. ICI immunotherapies alone or in combination with classical therapeutic approaches (e.g., chemotherapy and radiotherapy) are assigned as first-line or second-line treatment for approximately 50 cancer types (37). CTLA-4 inhibitors, ipilimumab and tremelimumab, received approval for use in different cancers, including colorectal cancer (CRC), melanoma, mesothelioma, NSCLC and RCC (38). PD-1/PD-L1-targeting antibodies have become some of the most commonly prescribed cancer treatments. Anti-PD-L1 antibodies avelumab and durvalumab received approval for the treatment of RCC, urothelial carcinoma, advanced bladder cancer and NSCLC (39). Atezolizumab is an anti-PD-L1 antibody used in the treatment of advanced bladder cancer and urothelial cancer (40, 41). The monoclonal antibody targeting PD-1, nivolumab, could be used in the treatment of several advanced cancers, such as Hodgkin’s lymphoma, melanoma, NSCLC, RCC, squamous head and neck cancer and urothelial carcinoma (42). The anti-PD-1 antibody pembrolizumab combined with lenvatinib was approved in treating patients with bladder cancer and endometrial cancer (EC) (43, 44). It is worth noting that a significant proportion of cancer patients receiving immunotherapy do not derive clinical benefit (45). Further development of alternative immune checkpoint-targeting agents is thus critical. The full understanding of the sophisticated mechanisms behind cancer immune escape will hopefully facilitate identification of more effective immunotherapeutic approaches to treat cancer.

## 3 Mechanisms of action of noncoding RNAs in cancer immune resistance

### 3.1 MicroRNAs

#### 3.1.1 Coordination of immune cell function

**Figure 2:**
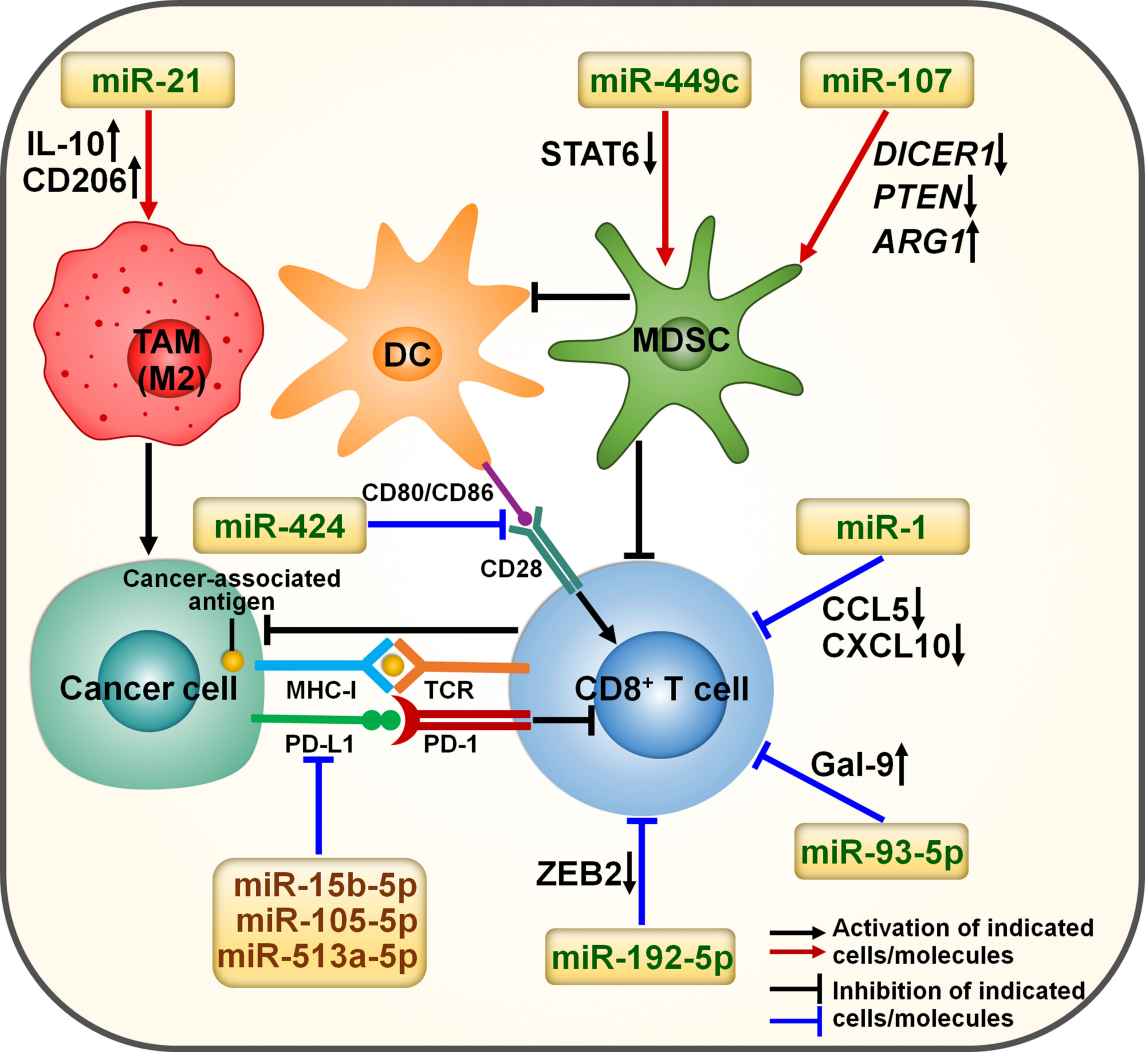
miRNA involvement in immunotherapy resistance in cancer. miRNAs can affect the efficacy of immunotherapy by orchestrating the tumor immune microenvironment. Cancer-derived exosomal miR-21 elevates the expression of IL-10 and CD206, thus promoting M2-like macrophage polarization. Exosomal miR-424 can suppress the CD28-CD80/86 costimulatory signaling, contributing to the impairment of T cell function. miR-449c promotes the expansion of mo-MDSCs by downregulating STAT6. Exosomal miR-107 induces the expansion and activation of MDSCs by regulating *DICER1*, *PTEN* and *ARG1*. miR-1 represses the migration of CD8^+^ T cells by reducing the expression of CCL5 and CXCL10. miR-15b-5p, miR-105-5p and miR-513a-5p may promote the activation of CD8^+^ T cells by targeting PD-L1. miR-192-5p restrains the cytotoxic activity of CD8^+^ T cells by downregulating ZEB2. miR-93-5p induces CD8^+^ T cell exhaustion by fostering Gal-9 augmentation. IL-10, interleukin-10; STAT6, signal transducer and activator of transcription 6; DICER1, double-stranded RNA-specific endoribonuclease 1; PTEN, phosphatase and tensin homolog; ARG1, arginase 1; TAM, tumor-associated macrophage; DC, dendritic cell; MDSC, myeloid-derived suppressive cell; MHC-I, major histocompatibility complex class I; TCR, T cell receptor; PD-L1, programmed cell death-ligand 1; PD-1, programmed cell death protein-1; CCL5, C-C motif chemokine ligand 5; CXCL10, C-X-C motif chemokine ligand 10; ZEB2, zinc finger E-box binding homeobox 2; Gal-9, galectin-9.

Immunosuppressive cells including myeloid-derived suppressive cells (MDSCs) and tumor-associated macrophages (TAMs) are crucial factors associated with immune resistance (46). Cytokines and signals released by immunosuppressive cells can foster cancer progression and immune evasion. Targeting immunosuppressive cells is a promising therapy to overcome immune resistance. miRNAs may affect the therapeutic effects of immunotherapy by regulating the activity of immunosuppressive cells (**Figure 2**). The expression level of miR-449c was increased in myeloid progenitor cells of melanoma-bearing mice in response to activation of C-X-C motif chemokine receptor 2 (CXCR2) (47). Overexpressed miR-449c promoted the expansion of monocytic MDSCs (mo-MDSCs) by downregulating signal transducer and activator of transcription 6 (STAT6) (**Table 1**). Conversely, deficiency of miR-449c repressed differentiation of myeloid progenitor cells into mo-MDSCs. MDSCs are found to play a role in blunting antitumor immunity and enhancing cancer resistance to immunotherapy (58). Thus, it is necessary to verify whether miR-449c can regulate melanoma cell response to immunotherapy.

**Table 1:** Representative miRNAs involved in immunotherapy resistance.

Regulatory Function	miRNA	Target	Action	Effect on immunotherapy resistance	Cancer type	Reference
**Tumor microenvironment**	miR-449c	STAT6	Promote mo-MDSC expansion	Promotion	Melanoma	(47)
	miR-107	*DICER1*, *PTEN* and *ARG1*	Promote MDSC expansion and activation	Promotion	Gastric cancer	(48)
	miR-21	IL-10 and CD206	Promote M2-like macrophage polarization	Promotion	Endometrial cancer	(49)
	miR-1	CCL5 and CXCL10	Inhibit CD8^+^ T cell migration	Promotion	Lung adenocarcinoma	(50)
	miR-93-5p	Gal-9	Induce CD8^+^ T cell dysfunction	Promotion	Hepatocellular carcinoma	(51)
	miR-424	CD28-CD80/86	Induce T cell dysfunction	Promotion	Colorectal cancer	(52)
	miR-192-5p	ZEB2	Repress CTL function	Promotion	Melanoma	(53)
**Immune checkpoint**	miR-15b-5p	PD-L1	Facilitate CD8^+^ T cell recruitment	Inhibition	Colorectal cancer	(54)
	miR-105-5p	PD-L1	Facilitate CD8^+^ T cell activation	Undetermined	Gastric cancer	(55)
	miR-513a-5p	PD-L1	Support cancer progression	Promotion	Hepatocellular carcinoma	(56)
**Intracellular signaling pathways**	miR-21-3p	TXNRD1	Promote IFN-γ-mediated ferroptosis	Inhibition	Melanoma	(57)

Exosomes are small membrane-enclosed vesicles released by diverse cell types and function as key regulators in cell-to-cell communication via transmitting diverse constituents (e.g., DNAs, lipids, ncRNAs and proteins) (59). Gastric cancer-released exosomes transported miR-107 to MDSCs (48). Exosomal miR-107 promoted the expansion and activation of MDSCs by downregulating tumor suppressors double-stranded RNA-specific endoribonuclease 1 (*DICER1*) and phosphatase and tensin homolog (*PTEN*) while upregulating arginase 1 (*ARG1*). Tumor-derived miR-107 mediated the communication between cancer cells and MDSCs, leading to tumor development and enhancement of immunotherapy resistance. EC cells facilitated monocyte transformation to M2-like TAMs under hypoxic condition (49). Mechanistically, hypoxic EC cells secreted miR-21-containing exosomes, which were taken up by monocytes. Exosome-derived miR-21 increased the expression of interleukin-10 (IL-10) and CD206, hence promoting M2-like macrophage polarization. Exosomal miR-21 reshaped the tumor immune microenvironment, which potentially facilitated EC development. Collectively, miRNAs act as mediators in tumor immune evasion. However, the exact effect of these miRNAs on the anticancer activity of immunotherapy should be further determined.

Cytotoxic CD8^+^ T cells function to induce apoptosis and efficiently eradicate cancer cells via direct cellular contacts (60, 61). CD8^+^ T cells have been recognized as the most powerful effectors in antitumor immunity and are the mainstay of successful cancer immunotherapy. Exposure to epidermal growth factor receptor-tyrosine kinase inhibitor (EGFR-TKI) treatment increased miR-1 expression in patients with lung adenocarcinoma (LUAD) (50). The cytokines C-C motif chemokine ligand 5 (CCL5) and C-X-C motif chemokine ligand 10 (CXCL10) can induce migration of intratumoral CD8^+^ T cells in cancer (62). Upregulated miR-1 inhibited the therapeutic effect of EGFR-TKI and restricted the migration of CD8^+^ T cells by downregulating CCL5 and CXCL10. miR-1 could be useful in identifying EGFR-TKI-resistant patients who may benefit from immunotherapy. Forced expression of miR-1 may be a mechanism underlying resistance to immunotherapy (e.g., ICI) in LUAD patients. miR-93-5p functioned as a critical initiating oncogene during hepatocarcinogenesis (51). Galectin-9 (Gal-9) causes the inactivation of various immune cells, such as natural killer (NK) cells and T cells, enabling cancer cells to evade from immunosurveillance (63, 64). miR-93-5p induced infiltrated CD8^+^ T cell dysfunction by favoring Gal-9 augmentation. Anti-Gal-9 antibodies reversed anti-PD-1 therapy resistance in a miR-93-5p-induced hepatocellular carcinoma (HCC) mouse model. The clinical benefit of targeting miR-93-5p and its target Gal-9 in HCC awaits further verification.

Hypoxia caused the enrichment of miR-424 in CRC-derived exosomes, which were subsequently transferred to tumor-infiltrating dendritic cells (DCs) and T cells (52). Exosome-delivered miR-424 impeded the CD28-CD80/86 costimulatory signaling in these immune cells, contributing to the formation of an immunosuppressive tumor microenvironment. Deficiency of tumor-derived miR-424 suppressed CRC development in a murine tumor model. In contrast, intratumoral inoculation of miR-424-containing exosomes accelerated tumor growth by blunting CD28-CD80/86-dependent antitumor immunity. The CD28-CD80/86 costimulatory pathways could repress PD-1-mediated T cell dysfunction and positively correlated with patient response to anti-PD-1 treatment (65–67). It was thus inferred that miR-424 was able to affect immunotherapy response in CRC. Consistently, specifically blocking tumor-derived functional miR-424 restored adaptive antitumor immunity and re-sensitized CRC cells to anti-PD-1/CLTA-4 treatment. Modification of immunosuppressive miRNA-containing exosomes could be developed as an adjuvant treatment for ICI-resistant CRC. Substantial research efforts should be made to comprehensively explore immune-related miRNAs in cancer cells and their derived exosomes.

Gap junctional shuttling of ncRNAs represents a vital mechanism of immunosuppression. miR-192-5p was strikingly enriched in hypoxic melanoma cells (53). The connexin-43 (Cx43)-constituted gap junctions mediated the transfer of miR-192-5p to DCs and melanoma-specific cytotoxic T lymphocytes (CTLs). Moreover, the delivery of miR-192-5p inhibited the cytotoxic activity of CTLs and contributed to cancer immune escape by downregulating zinc finger E-box binding homeobox 2 (ZEB2), a transcription factor that modulated the expression of terminal effector genes in CD8^+^ T cells. Altogether, these findings may provide new opportunities for the development of anticancer immunotherapies aimed to improve clinical outcomes of cancer patients.

#### 3.1.2 Regulation of inhibitory immune checkpoint-mediated immune evasion

The PD-1/PD-L1-based pathway has become a pivotal immune checkpoint in recent years and is a key target for cancer immunotherapy. However, emerging evidence indicates that the PD-1/PD-L1 pathway has opposite effects on immunotherapy efficacy. On the one hand, reduction of PD-L1 expression via genetic or pharmacological inhibition reinforces the efficacy of anti-PD-1/PD-L1 therapy. The expression level of miR-15b-5p gradually reduced with the development of microsatellite stable (MSS) CRC (54). The protein level of PD-L1 showed an opposite trend. Further study demonstrated that overexpressed miR-15b-5p prevented CRC tumorigenesis and sensitized cancer cells to PD-1 blockade by downregulating PD-L1 at the posttranscriptional level *in vivo*. Conversely, depletion of miR-15b-5p supported CRC tumorigenesis by upregulating PD-L1 and repressing the recruitment of CD8^+^ T cells. Increasing miR-15b-5p expression enhanced the efficacy of ICI therapy. The therapeutic effects of combined miR-15b-5p and PD-1 blockade should be verified in further clinical studies. Likewise, miR-105-5p promoted the activation of CD8^+^ T cells in gastric cancer by directly targeting PD-L1 (55). The expression of miR-105-5p was induced by the hypomethylation of a cancer- and germline-specific promoter of its host gene, *GABRA3*. miR-105-5p may be a target for gastric cancer immunotherapy, which warrants follow-up studies.

On the other hand, downregulation of PD-L1 mediates the resistant response to ICI (68). miR-513a-5p promoted proliferation, invasion and migration of HCC cells by reducing PD-L1 expression, leading to increased resistance to anti-PD-1 therapy in HCC (56). Specifically, β-glucuronidase-mediated inhibition of miR-513a-5p inhibited HCC progression and improved the efficacy of anti-PD-1 treatment in murine tumor model. Antagonism of miR-513a-5p may be an effective approach for improving the therapeutic potential of anti-PD-1 treatment. The addition of ncRNA-based therapies targeting a large variety of immune checkpoints would pave the way for surmounting the resistance to immunotherapy and enable better clinical outcomes in cancer patients.

#### 3.1.3 Modulation of cell death pathways

**Figure 3:**
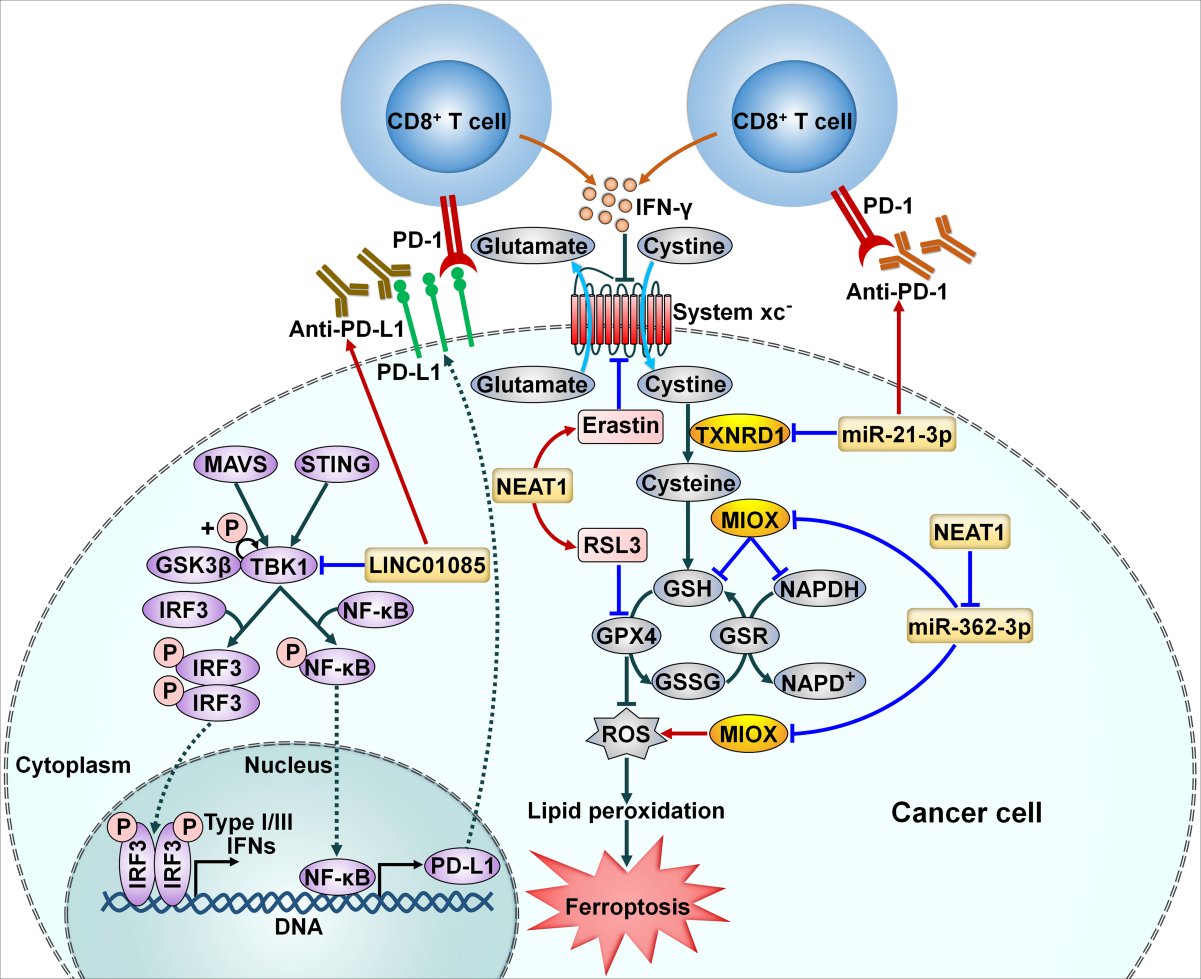
Implication of ncRNAs in cellular signaling pathways related to cancer immune resistance. NcRNAs can affect the efficacy of immunotherapy through modulation of cancer cell ferroptosis. miR-21-3p accelerates IFN-γ-mediated ferroptosis by downregulating TXNRD1 to impel lipid peroxidation. NEAT1 increases MIOX expression by sponging miR-362-3p. This event enhances ROS production and decreases the intracellular levels of NADPH and GSH, eventually leading to cancer cell ferroptosis. Moreover, NEAT1 reinforces the anticancer potency of ferroptosis activators erastin and RSL3. LINC01085 inhibits the phosphorylation of TBK1 by abating its interaction with GSK3β. Accordingly, LINC01085 blocks the activation of the STING/MAVS/IRF3 signaling pathway, resulting in downregulation of NF-κB and PD-L1 and decreased production of type I/III IFNs. LINC01085 can act in synergy with anti-PD-L1 treatment. PD-L1, programmed cell death-ligand 1; PD-1, programmed cell death protein-1; IFN-γ, interferon-γ; MAVS, mitochondrial antiviral signaling protein; STING, stimulator of interferon genes; GSK3β, glycogen synthase kinase 3β; TKB1, TANK-binding kinase 1; IRF3, interferon regulatory factor 3; NF-κB, nuclear factor-κB; TXNRD1, thioredoxin reductase 1; RSL3, RAS-selective lethal 3; MIOX, myo-inositol oxygenase; GSH, glutathione; NAPDH, nicotinamide adenine dinucleotide phosphate; GPX4, glutathione peroxidase 4; GSR, glutathione reductase; GSSG, oxidized glutathione; ROS, reactive oxygen species.

Ferroptosis, an iron-dependent form of regulated necrosis, is mainly initiated by the lethal accumulation of lipid peroxidation catalyzed by cellular labile free iron (69). Cancer cell ferroptosis induced by interferon-γ (IFN-γ) released by tumor-infiltrating CD8^+^ T cells potentiates the efficacy of immunotherapy (70). The involvement of miRNAs in ferroptosis suggests their regulatory effects on the therapeutic effect of immunotherapy. Thioredoxin reductase 1 (TXNRD1) acts as a negative regulator of ferroptosis (71). miR-21-3p promoted IFN-γ-mediated ferroptosis by downregulating TXNRD1 to impel lipid peroxidation (57) (**Figure 3**). Importantly, miR-21-3p overexpression sensitized melanoma cells to anti-PD-1 therapy through promotion of ferroptosis. Nanoparticle delivery of miR-21-3p was proven to synergize with anti-PD-1 antibody without obvious adverse effects in a preclinical mouse model. Nanoparticle delivery of ferroptosis-related ncRNAs may have translational potential in cancer immunotherapy, which deserves more detailed investigations.

### 3.2 Long noncoding RNAs

#### 3.2.1 Blockade of antigen presentation

**Figure 4:**
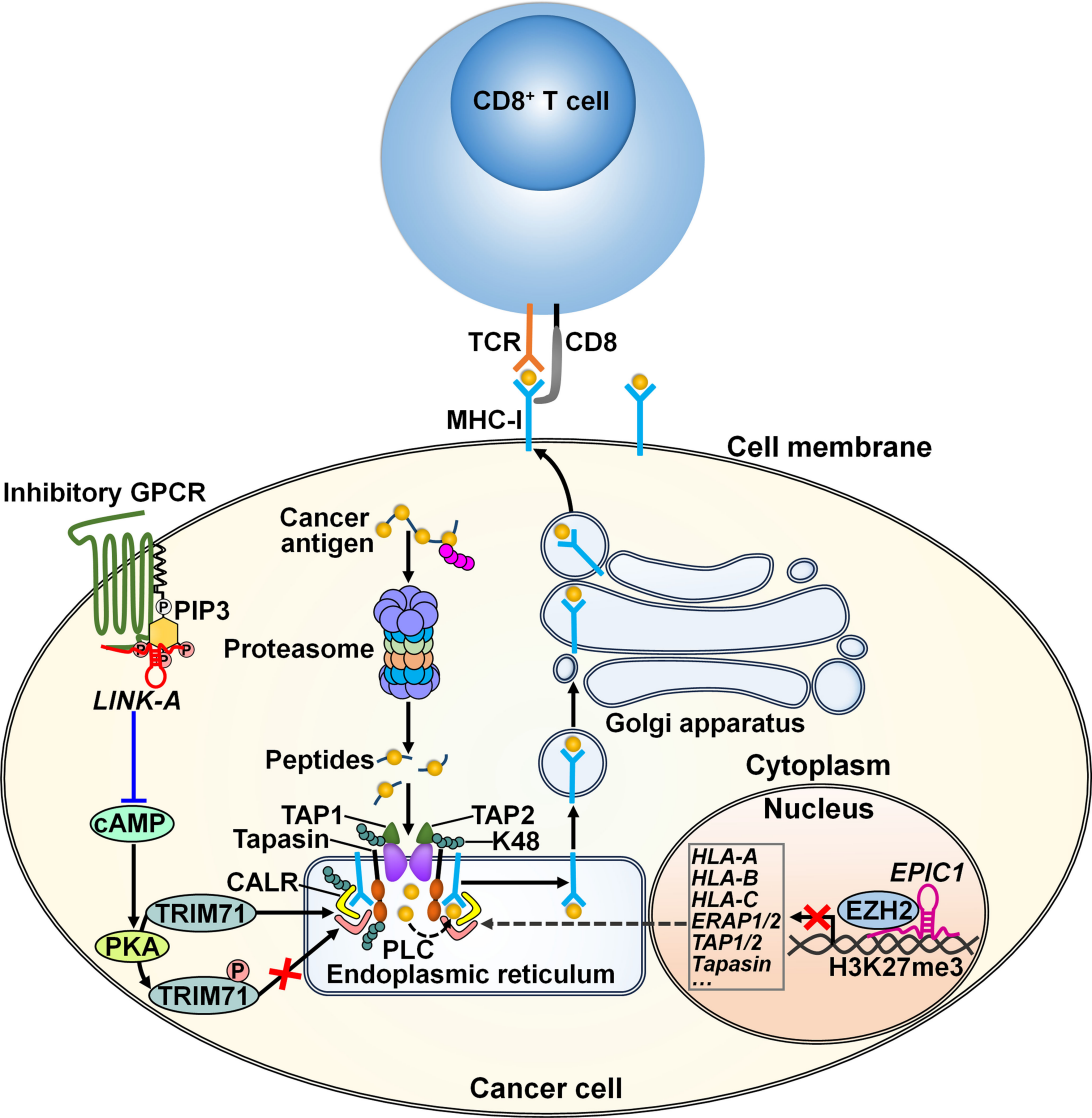
Regulatory mechanisms of lncRNAs in cancer cell antigen presentation. The lncRNA *LINK-A* supports the interaction between inhibitory GPCR and PIP3 pathways, leading to downregulation of cAMP and consequent inhibition of PKA-mediated TRIM71 phosphorylation. As a result, *LINK-A* promotes K48-polyubiquitination-mediated degradation of the PLC components including tapasin, TAP1, TAP2, and CALR, constituting a resistance strategy for immunological cancer escape. The linRNA *EPIC1* causes the epigenetic silencing of antigen presentation genes (e.g., *HLA-A*, *HLA-B*, *HLA-C*) and antigen processing genes (e.g., *ERAP1/2*, *TAP1/2* and *tapasin*) through interaction with EZH2. These lncRNAs promotes cancer resistance to immunotherapy. TCR, T cell receptor; MHC-I, major histocompatibility complex class I; GPCR, G-protein-coupled receptor; PIP3, phosphatidylinositol- (3,4,5)-triphosphate; cAMP, cyclic adenosine monophosphate; PKA, protein kinase A; TRIM71, tripartite motif-containing 71; TAP1, transporter associated with antigen processing 1; TAP2, transporter associated with antigen processing 2; CALR, calreticulin; PLC, peptide-loading complex; HLA-A, human leukocyte antigen A; HLA-B, human leukocyte antigen B; HLA-C, human leukocyte antigen C; ERAP1/2, endoplasmic reticulum aminopeptidase 1/2; EZH2, zeste homolog 2.

LncRNAs play important roles in cancer immunotherapy resistance. Disruption of the antigen presentation pathway forms a significant mechanism of cancer resistance to immunotherapy. LncRNAs are vital regulators of the antigen presentation process (**Figure 4**). The lncRNA *LINK-A* exhibited a tissue-specific expression pattern in mammary glands (72). This lncRNA promoted the interaction between phosphatidylinositol- (3,4,5)-triphosphate (PIP3) and inhibitory G-protein-coupled receptor (GPCR) pathways, resulting in the inactivation of the cyclic adenosine monophosphate (cAMP)/protein kinase A (PKA) signaling pathway (**Table 2**). This led to the inhibition of PKA-mediated phosphorylation of an E3 ubiquitin ligase tripartite motif-containing 71 (TRIM71). Hypophosphorylated TRIM71 facilitated K48-linked polyubiquitination and proteasome-mediated degradation of the antigen peptide-loading complex (PLC) components (e.g., tapasin, transporter associated with antigen processing 1 (TAP1), TAP2, and calreticulin (CALR)) and inherent tumor suppressors Rb and p53. Thus, *LINK-A* acted to attenuate intrinsic tumor suppressor barriers via the GPCR/PKA/TRIM71 signaling cascade, forming a resistance strategy for immunological cancer escape. Knockdown of *LINK-A* markedly improved the protein stability of the PLC components and MHC class I (MHC-I) complex and enhanced the infiltration of CD8^+^ T cells into the tumor tissue, hence sensitizing breast cancer cells to ICI.

**Table 2:** Representative lncRNAs associated with cancer immune response.

Regulatory Function	lncRNA	Action	Mechanism	Effect on immunotherapy resistance	Cancer type	Reference
**Tumor antigen presentation**	*LINK-A*	Induce degradation of the antigen PLC components	Promote the association of PIP3 with GPCR	Promotion	Breast cancer	(72)
	*EPIC1*	Inhibit the expression of antigen presentation genes and antigen processing genes	Interact with EZH2	Promotion	Breast cancer	(73)
**Tumor microenvironment**	SNHG14	Suppress CD8^+^ T cell activity	Act as a molecular sponge for miR-5590-3p	Promotion	Diffuse large B cell lymphoma	(74)
	LINC00261	Correlate with decreased Treg infiltration and increased CTL infiltration	Undetermined	Inhibition	Lung adenocarcinoma	(75)
	LINC01132	Inhibit the intratumoral infiltration of CD8^+^ T cells	Interfere with NRF1 binding to DDP4	Promotion	Hepatocellular carcinoma	(76)
	*PVT1*	Reduce the intratumoral infiltration of CD8^+^ T cells	Foster TDO2 phosphorylation and elevate kynurenine level	Promotion	Pancreatic ductal adenocarcinoma	(77)
**Intracellular signaling pathways**	NEAT1	Activate MIOX-mediated ferroptosis	Act as a molecular sponge for miR-362-3p	Undetermined	Hepatocellular carcinoma	(78)
	LINC01085	Downregulate NF-κB and PD-L1; reduce IFN production	Inhibit the interaction between TBK1 and GSK3β	Inhibition	Prostate cancer	(79)

The long intergenic ncRNA (linRNA) *EPIC1* could interact with the histone methyltransferase enhancer of zeste homolog 2 (EZH2), resulting in the epigenetic silencing of antigen presentation genes (e.g., human leukocyte antigen A (*HLA-A*), *HLA-B*, *HLA-C*), antigen processing genes (e.g., endoplasmic reticulum aminopeptidase 1/2 (*ERAP1/2*), *TAP1/2* and *tapasin*) as well as the IFN-γ receptor *IFNGR1* (73). Consequently, *EPIC1* induced breast cancer resistance to ICI therapy through inhibition of antigen presentation and the IFN-γ/Janus kinase (JAK)/STAT1 pathways. Oppositely, knockdown of EZH2 counteracted the tumor-promoting effect of *EPIC1*. The linRNA *EPIC1* may serve as a potential target for cancer immunotherapy. The mechanisms by which *EPIC1* regulates the interaction between EZH2 and its target genes warrant further study.

#### 3.2.2 Regulation of T cell activity

**Figure 5:**
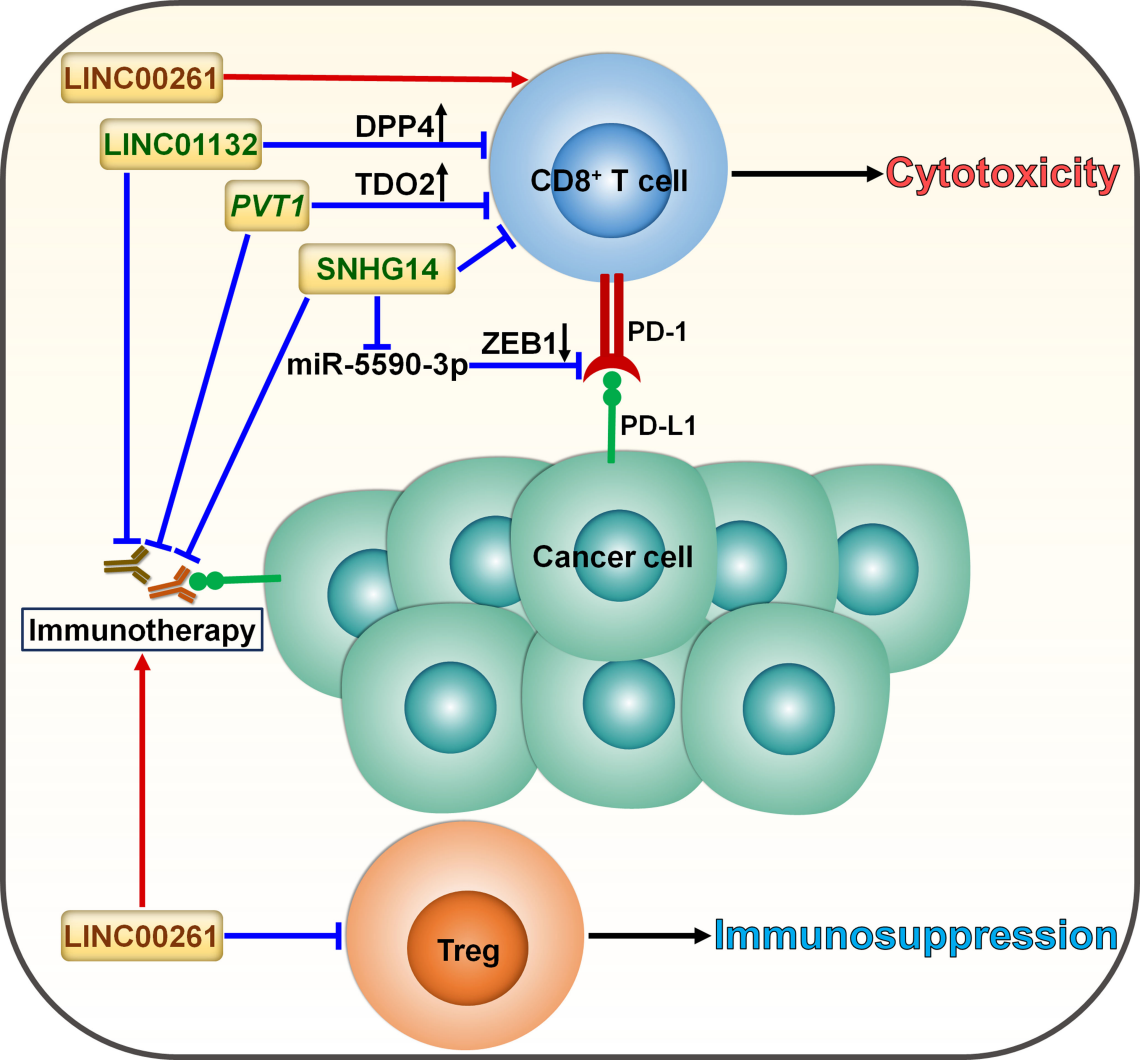
Roles of lncRNAs in skewing T cell balance in cancer. LncRNAs serve as momentous factors influencing the CD8^+^ T cell/Treg equilibrium. LINC01132 impairs the intratumoral infiltration of CD8^+^ T cells to compromise antitumor immunity by promoting DPP4 expression. *PVT1* inhibits the infiltration and cytotoxicity of CD8^+^ T cells by provoking TDO2. SNHG14 restricts CD8^+^ T cell activity and accelerates cancer progression via ZEB1-mediated activation of PD-1/PD-L1 immune checkpoint by sponging miR-5590-3p. These lncRNAs facilitate cancer immune evasion and dampen the effectiveness of immunotherapy through induction of T cell dysfunction. LINC00261 suppresses the expansion of tumor-promoting Tregs and tips the balance toward tumor suppression, potentially strengthening the tumor-killing effect of immunotherapy. DPP4, dipeptidyl peptidase 4; TDO2, tryptophan 2,3-dioxygenase; ZEB1, zinc finger E-box binding homeobox 1; PD-1, programmed cell death protein-1; PD-L1, programmed cell death-ligand 1; Treg, regulatory T cell.

Cancer immunotherapy predominantly relies on either activating or restoring the function of CD8^+^ T cells (80). LncRNAs can coordinate the activation of T cells and thus affect the immunotherapy response (**Figure 5**). LncRNA small nucleolar RNA host gene 14 (SNHG14) served as a tumor promoter in diffuse large B cell lymphoma (DLBCL) (74). SNHG14 increased ZEB1 expression to activate PD-1 by sponging miR-5590-3p. Consequently, SNHG14/miR-5590-3p repressed CD8^+^ T cell activity and promoted DLBCL progression through ZEB1-mediated activation of PD-1/PD-L1 immune checkpoint. Particularly, the supplementation of anti-PD-1 or anti-PD-L1 reversed the effect of SNHG14 overexpression on CD8^+^ T cell activity. Depletion of SNHG14 potentially blocks immune evasion and enhances the effectiveness of immunotherapy in DLBCL through regulating the PD-1/PD-L1 axis.

Cyclin-dependent kinase 1 (CDK1)-related lncRNA, LINC00261, was significantly downregulated in LUAD compared with normal tissues and was correlated with poor prognosis in LUAD (75). The level of IL-8, mainly derived from macrophages, dramatically elevated in ICI-resistant patients, which could be ascribed to the downregulation of LINC00261 in LUAD. These results suggested the interrelation between the LINC00261 level and immunotherapy resistance in LUAD. In addition, reduced expression of LINC00261 was likely to correlate with increased infiltration of regulatory T cells (Tregs) and decreased infiltration of cytotoxic T cells, eventually leading to immunosuppression. Enforced expression of LINC00261 may improve the effectiveness of immunotherapy, which is worthy of further study. Concerted research efforts are required to reveal the mechanisms through which LINC00261 affects the responsiveness to immunotherapy in LUAD.

The expression level of LINC01132 was remarkably higher in HCC tissues than normal tissues and correlated with poor prognosis of HCC patients (76). LINC01132 increased the expression of dipeptidyl peptidase 4 (DPP4) by competitively binding to nuclear factor erythroid 2-related factor 1 (NRF1). Depletion of LINC01132 promoted the intratumoral infiltration of CD8^+^ T cells and potentiated the efficacy of anti-PD-L1 therapy in HCC-bearing mice via DPP4 inhibition.

Pancreatic ductal adenocarcinoma (PDAC)-produced IL-6 induced the expression of the lncRNA plasmacytoma variant translocation 1 (*PVT1*) in tumor-associated nonmyelinating Schwann cells (TASc) (77). Tryptophan 2,3-dioxygenase (TDO2), an important enzyme in the kynurenine pathway, fostered cancer progression by inducing CD8^+^ T cell dysfunction (81). *PVT1* fostered TDO2 phosphorylation and enhanced its enzymatic activities in the conversion of tryptophan to kynurenine. As a result, *PVT1* reduced CD8^+^ T cell infiltration and promoted PDAC carcinogenesis (77). TASc inhibition significantly suppressed PDAC growth and enhanced the intratumoral infiltration and cytotoxicity of CD8^+^ T cells. Reportedly, high levels of kynurenine contribute to immunotherapy resistance in cancer patients (82). As expected, TASc inhibition synergized with anti-PD-1 antibody in PDAC-bearing mice, which could be partially attributable to reduced kynurenine production. It will be important to validate the direct effect of *PVT1* on the status of the TME and the efficacy of anti-PD-1 antibody in further studies.

#### 3.2.3 Modulation of cellular signaling pathways

LncRNAs have been implicated in the regulation of multiple pathways through diverse genetic or epigenetic mechanisms (83). LncRNA nuclear paraspeckle assembly transcript 1 (NEAT1) increased the expression of myo-inositol oxygenase (MIOX) in HCC by sponging miR-362-3p (78) (**Figure 3**). MIOX facilitated the production of reactive oxygen species (ROS) and reduced the intracellular levels of nicotinamide adenine dinucleotide phosphate (NADPH) and glutathione (GSH), thus promoting ferroptosis. *In vitro* and *in vivo* experiments manifested that upregulation of NEAT1 strengthened the anticancer potency of ferroptosis activators erastin and RAS-selective lethal 3 (RSL3) through promotion of ferroptosis. Inducing ferroptosis via NEAT1 overexpression could serve as a prospective therapeutic method for HCC immunotherapy.

LINC01085 was deleted in docetaxel-resistant castration resistant prostate cancer (CRPC) and predicted favorable outcomes of CRPC patients (79). It could competitively interact with TANK-binding kinase 1 (TBK1) and glycogen synthase kinase 3β (GSK3β), hence abating the affinity of TBK1 and GSK3β and reducing TBK1 phosphorylation at the Ser-172 site. These events inhibited the activation of the stimulator of interferon genes (STING)/mitochondrial antiviral signaling protein (MAVS)/IFN regulatory factor 3 (IRF3) signaling pathway, contributing to downregulation of nuclear factor-κB (NF-κB) and PD-L1 as well as reduced production of type I/III IFNs. *In vivo* experiments indicated that overexpression of LINC01085 in combination with anti-PD-L1 treatment produced a significant tumor-antagonizing effect than anti-PD-L1 treatment alone. Overall, this study provided a scientific rationale to combine LINC01085 agonists with ICI therapy to enhance the therapeutic effect for CRPC in the future.

### 3.3 Circular RNAs

#### 3.3.1 Induction of immune cell dysfunction

**Figure 6:**
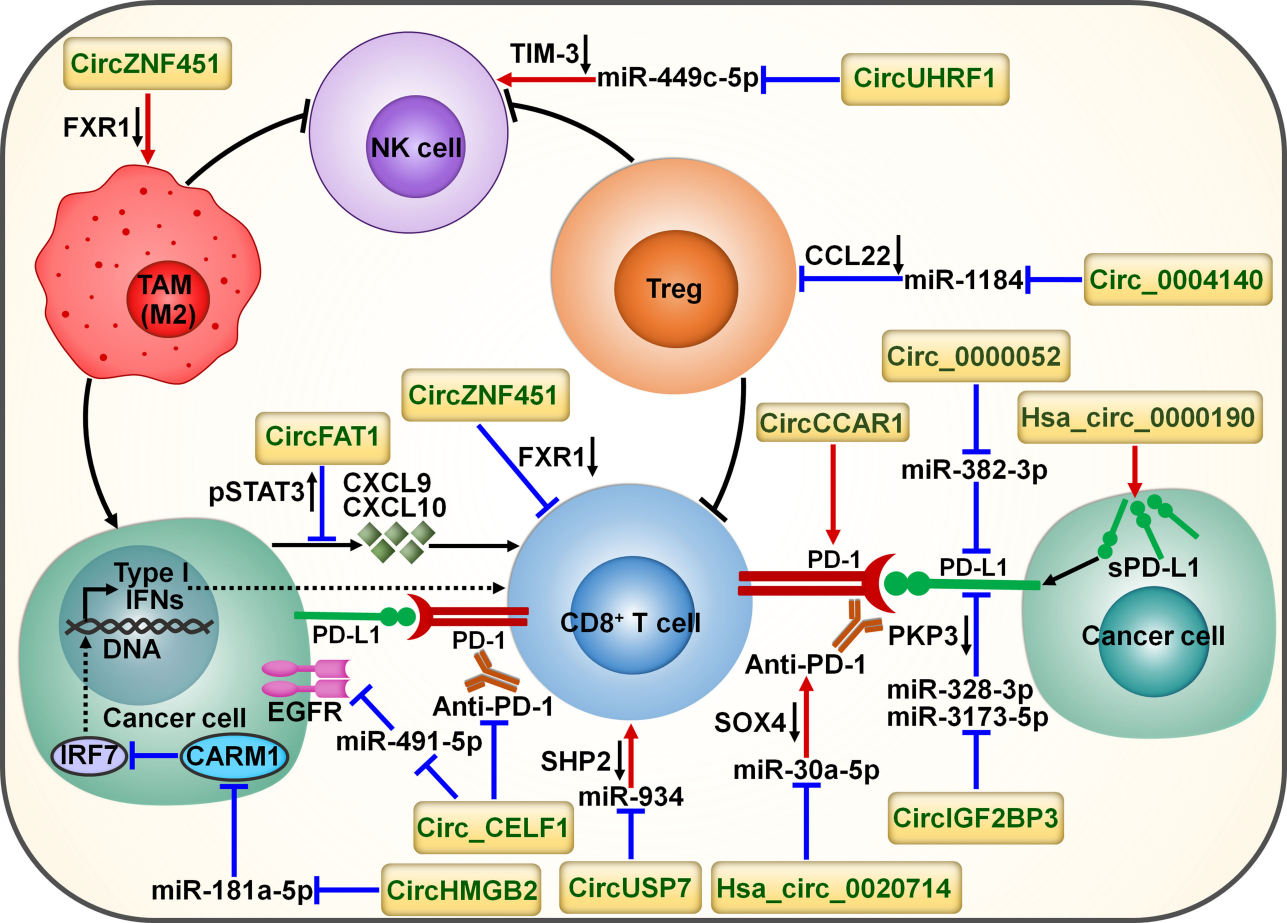
Schematic representation of the interplay between circRNAs and tumor immune microenvironment. Cancer-derived exosomal circZNF451 mediates the degradation of FXR1 in macrophages. It can reshape the immunosuppressive tumor microenvironment by driving macrophage polarization toward the M2-like phenotype as well as dysfunction of cytotoxic CD8^+^ T cells. Cancer-secreted exosomal circUHRF1 restrains NK cell ability by targeting miR-449c-5p to increase TIM-3 expression. CircUHRF1 attenuates cancer cell sensitivity to anti-PD-1 treatment. Circ_0004140 increases the expression of CCL22 to recruit Tregs in the tumor microenvironment by directly targeting miR-1184. CircFAT1 exerts an inhibitory action on CD8^+^ T cell activation by stimulating STAT3 to block the secretion of IFN-I-induced chemokines (e.g., CXCL9 and CXCL10). Cancer-secreted exosomal circUSP7 restricted CD8^+^ T cell function by regulating the miR-934/SHP2 axis. Circ_CELF1 upregulates the oncogene EGFR by acting as a ceRNA for miR-491-5p. Circ_CELF1 can abrogate the anticancer effects of anti-PD-1 therapy. CircHMGB2 increases CARM1 expression by sponging miR-181a-5p, thereby limiting the IFN-I response. Thus, circHMGB2 plays a critical role in the molding of an immunosuppressive tumor microenvironment. CircRNAs are implicated in cancer immune escape by controlling the immune checkpoints. CircCCAR1 contributes to CD8^+^ T cell dysfunction by stabilizing PD-1. Circ_0000052 directly combines with miR-382-3p to relieve its inhibition of PD-L1. Hsa_circ_0000190 can elevate the expression of sPD-L1 and regulate immunotherapy response. Hsa_circ_0020714 diminishes cancer sensitivity to anti-PD-1 treatment by sponging miR-30a-5p to upregulate SOX4. CircIGF2BP3 prevents proteasome-mediated degradation of PD-L1 by targeting miR-328-3p and miR-3173-5p to upregulate PKP3. FXR1, fragile X-related protein 1; NK cell, natural killer cell; TIM-3, mucin domain-containing protein-3; TAM, tumor-associated macrophage; Treg, regulatory T cell; CCL22, C-C motif chemokine ligand 22; pSTAT3, phosphorylated signal transducer and activator of transcription 3; CXCL9, C-X-C motif chemokine ligand 9; CXCL10, C-X-C motif chemokine ligand 10; IFN, interferon; PD-1, programmed cell death protein-1; PD-L1, programmed cell death-ligand 1; sPD-L1, soluble programmed cell death-ligand 1; PKP3, plakophilin 3; EGFR, epidermal growth factor receptor; SOX4, Sry-related high-mobility group box 4; IRF7, interferon regulatory factor 7; CARM1, coactivator-associated arginine methyltransferase 1; SHP2, Src homology 2 domain-containing phosphatase 2.

CircRNAs have emerged as key factors that act in an entangled network of interactions with diverse miRNAs or proteins to remodel the tumor immune microenvironment (**Figure 6**). CircUHRF1 was upregulated in HCC tissues compared with matched adjacent normal tissues, and its high expression indicated NK cell exhaustion and poor prognosis in HCC patients (84). CircUHRF1 was also highly expressed in HCC cell-derived exosomes. T cell immunoglobulin and mucin domain-containing protein-3 (TIM-3) is a major inhibitory receptor on NK cells (85), and NK cells with elevated TIM-3 expression displayed a decreased capacity to initiate antitumor immune responses (86). HCC-secreted exosomal circUHRF1 could be delivered into NK cells, where it restricted NK cell ability to secrete IFN-γ and tumor necrosis factor-α (TNF-α) by targeting miR-449c-5p to enhance TIM-3 expression (84) (**Table 3**). CircUHRF1 supported HCC development in a NK cell- and exosome-dependent manner. CircUHRF1 deficiency increased HCC cell sensitivity to anti-PD-1 treatment and improved the overall survival rate in a xenograft mouse model. Altogether, circUHRF1 mediates the crosstalk between HCC cells and NK cells, leading to the impairment of NK cell function through upregulation of TIM-3 expression. CircUHRF1 functions as a critical factor affecting resistance to anti-PD-1 treatment. Reduction of circUHRF1 expression in HCC may improve patient outcomes by rescuing NK cell function. Tumor-infiltrating CD8^+^ T cells also had high expression levels of TIM-3. It is intriguing whether exosomal circUHRF1 could induce T cell dysfunction by regulating the miR-449c-5p/TIM-3 pathway. The involvement of circUHRF1 in the communication between cancer cells and tumor microenvironment is worthy of continued study.

**Table 3:** Overview of reported immune resistance-related circRNAs in cancer.

Regulatory Function	CircRNA	Target	Action	Effect on immunotherapy resistance	Cancer type	Reference
**Tumor microenvironment**	CircUHRF1	miR-449c-5p/TIM-3	Induce NK cell exhaustion	Promotion	Hepatocellular carcinoma	(84)
	CircFAT1	STAT3	Repress the intratumoral infiltration of CD8^+^ T cells	Promotion	Head and neck squamous cell carcinoma	(87)
	CircZNF451	FXR1	Induce M2-like macrophage polarization and CD8^+^ T cell exhaustion	Promotion	Lung adenocarcinoma	(88)
	Circ_0004140	miR-1184/CCL22	Induce CTL dysfunction	Promotion	Lung adenocarcinoma	(89)
	CircUSP7	miR-934/SHP2	Restrain CD8^+^ T cell function	Promotion	Non-small cell lung cancer	(90)
**Immune evasion**	Circ_0000052	miR-382-3p/PD-L1	Promote HNSCC cell aggressiveness	Undetermined	Head and neck squamous cell carcinoma	(91)
	Hsa_circ_0000190	sPD-L1	Correlate with cancer progression	Promotion	Non-small cell lung cancer	(92)
	CircIGF2BP3	miR-328-3p/miR-3173-5p/PKP3	Block PD-L1 degradation; inhibit T cell immunity	Promotion	Non-small cell lung cancer	(93)
	Hsa_circ_0020714	miR-30a-5p/SOX4	Correlate with poor prognosis	Promotion	Non-small cell lung cancer	(94)
	CircCCAR1	miR-127-5p/WTAP; PD-1	Induce CD8^+^ T cell dysfunction	Promotion	Hepatocellular carcinoma	(95)
**Oncogenes**	Circ_CELF1	miR-491-5p/EGFR	Foster cancer development	Promotion	Non-small cell lung cancer	(96)
	CircHMGB2	miR-181a-5p/CARM1	Block the IFN-I response	Promotion	Non-small cell lung cancer	(97)

CircFAT1 was strikingly upregulated in head and neck squamous cell carcinoma (HNSCC) relative to matched normal tissues and its high expression indicated HNSCC metastasis and poor prognosis (87). CircFAT1 combined with STAT3 and facilitated its activation by reducing Src homology 2 domain-containing phosphatase 1 (SHP1)-mediated dephosphorylation of phosphorylated STAT3 (pSTAT3). pSTAT3 bound to STAT1 to block type I IFN (IFN-I) response (98). As expected, circFAT1 depletion enhanced the production of IFN-I-induced chemokines (e.g., CXCL9 and CXCL10) that were intertwined with recruitment of CD8^+^ T cells to the tumor site (99). Consistently, circFAT1 knockdown potentiated the efficacy of anti-PD-1 treatment *in vivo* by promoting CD8^+^ T cell infiltration into tumor tissues (87). Upregulation of circFAT1 represents a vital molecular mechanism, which contributes to the molding of an immunosuppressive microenvironment in HNSCC.

CircZNF451 was highly expressed in the exosomes of LUAD patients refractory to anti-PD-1 treatment (88). LUAD-derived exosomal circZNF451 could induce the ubiquitination of fragile X-related protein 1 (FXR1) via the E3 ligase TRIM56 in macrophages. CircZNF451-mediated FXR1 degradation abolished the inhibition on the downstream E74-like factor 4 (ELF4)/IRF4 signaling pathway. ELF4 plays an important role in both innate and adaptive immunity by affecting the development and activity of NK and CD8^+^ T cells (100). Accordingly, exosomal circZNF451 reshaped the immunosuppressive tumor microenvironment by fostering macrophage polarization toward an anti-inflammatory M2-like phenotype as well as dysfunction of cytotoxic CD8^+^ T cells. Overexpression of circZNF451 attenuated the sensitivity of anti-PD-1 therapy in a xenograft model, while conditional silencing of ELF4 in macrophages reversed this effect. Collectively, exosomal circZNF451 may act as a new indicator for immunotherapy resistance and a target to enhance the efficacy of PD-1 blockade in LUAD. Another LUAD-enriched circ_0004140 could support cancer cell proliferation and migration (89). Circ_0004140 elevated the expression of CCL22 by directly interacting with miR-1184. CCL22 can recruit Tregs in the tumor microenvironment (101). Tregs are able to suppress CTL proliferation and induce immune suppression. Thus, overexpression of circ_0004140 was associated with impairment of CTL function. Combining CCL22/C-C motif chemokine receptor 4 (CCR4) axis inhibitor and anti-PD-1 treatment reversed immune resistance and reduced LUAD progression.

The plasma levels of exosomal circUSP7 were higher in NSCLC patients than healthy controls and correlated with poor clinical prognosis and CD8^+^ T cell dysfunction in NSCLC (90). CircUSP7 in patient plasma was mainly secreted by NSCLC cells via exosomes. Exosomal circUSP7 suppressed the ability of CD8^+^ T cells to secrete granzyme B (GzmB), IFN-γ, perforin and TNF-α. SHP2 is a ubiquitous tyrosine phosphatase that affects diverse immune cell signaling cascades (102). Importantly, SHP2 negatively regulated CD8^+^ T cell proliferation and function. Exosomal circUSP7 restrained CD8^+^ T cell function by serving as a miR-934 sponge to increase SHP2 expression (90). The *in vivo* experiment showed that exosomal circUSP7 overexpression enhanced the resistance to anti-PD-1 treatment and promoted tumor progression in NSCLC-bearing humanized NSG mice through induction of CD8^+^ T cell exhaustion. Furthermore, NSCLC patients with high levels of exosomal circUSP7 showed an apparent phenotype of resistance to anti-PD-1 therapy. Altogether, exosomal circUSP7 played a critical role in promoting tumor immune escape and immunotherapy resistance via the miR-934/SHP2 axis in NSCLC. Peripheral blood exosomal circUSP7 could be used as a novel marker of anti-PD-1 treatment in NSCLC. Reduction of circUSP7 expression may be effective in restoring CD8^+^ T cell functionality and augmenting immunotherapy efficacy in NSCLC patients.

#### 3.3.2 Promotion of cancer immune evasion

The increased expression of PD-L1 on cancer cells restricts the function of tumor-infiltrating T cells through interaction with PD-1 loaded on T cells and contributes to an immunosuppressive microenvironment (36). Circ_0000052 directly interacted with miR-382-3p to relieve its inhibition of PD-L1 (91). The resultant upregulation of PD-L1 led to HNSCC cell aggressiveness. Hsa_circ_0000190 could enhance the expression of soluble PD-L1 (sPD-L1) in NSCLC (92). The expression level of sPD-L1 showed a positive linkage with disease progression in NSCLC patients following immunotherapy, hinting the crucial role of sPD-L1 in regulating immunotherapy response. Hsa_circ_0000190 might mediate NSCLC immune resistance through upregulation of sPD-L1. The mechanism by which hsa_circ_0000190 coordinates sPD-L1 expression remains to be deciphered.

NSCLC-enriched circIGF2BP3 showed a negative interrelation with the infiltration level of intratumoral CD8^+^ T cells (93). CircIGF2BP3 acted as a molecular sponge for miR-328-3p and miR-3173-5p to elevate plakophilin 3 (PKP3) expression. PKP3 enhanced the mRNA stability of the deubiquitinase ovarian tumor domain-containing ubiquitin aldehyde-binding protein 1 (OTUB1) by binding to FXR1, further protecting PD-L1 from proteasomal degradation. The circIGF2BP3/PKP3 regulatory axis accounted for cancer cell escape from immune surveillance by increasing PD-L1 expression. Accordingly, circIGF2BP3 dampened T cell-mediated antitumor immunity and accelerated NSCLC growth. PKP3 knockdown reinforced the therapeutic effect of PD-1 blockade in tumor-bearing mice.

High expression levels of hsa_circ_0020714 was correlated with resistance to anti-PD-1 immunotherapy and predicted a poor prognosis in NSCLC patients (94). Reportedly, the oncogene Sry-related high-mobility group box 4 (SOX4) favored immune escape and improved resistance to PD-1 blockade in cancer (103). Hsa_circ_0020714 induced NSCLC immune evasion and attenuated sensitivity to anti-PD-1 treatment by sponging miR-30a-5p to upregulate SOX4 (94). Blockade of the hsa_circ_0020714/miR-30a-5p/SOX4-related pathway may result in better immunotherapeutic effects in NSCLC.

CircCCAR1 was enriched in serum exosomes of HCC patients compared with those of healthy controls (95). CircCCAR1 acted as an oncogenic driver in HCC by sponging miR-127-5p to upregulate its target Wilms tumor 1-associated protein (WTAP). Moreover, exosomal circCCAR1 released by HCC cells was ingested by CD8^+^ T cells and resulted in CD8^+^ T cell dysfunction by stabilizing PD-1. Exosomal circCCAR1 released by HCC cells enhanced resistance to anti-PD-1 immunotherapy and reduced the survival time of HCC-bearing mice by retarding CD8^+^ T cell expansion and infiltration into tumor tissues. Modulation of PD-L1/PD-1-regulating circRNAs may represent an effective strategy to reverse T cell exhaustion and maximize immunotherapeutic effect in cancer patients. Nevertheless, clear evidence is needed to better understand the role of these circRNAs in modifying immunotherapy responsiveness.

#### 3.3.3 Upregulation of oncogenes

Oncogenes have been implicated in immune system dysfunction and thus affect the benefits of immune-based cancer treatments (104). CircRNAs counteract the anticancer efficacy of immunotherapy through modulation of oncogenes. The expression level of circ_CELF1 was higher in NSCLC tissues than their adjacent nontumor tissues (96). Circ_CELF1 increased the expression of the oncogene epidermal growth factor receptor (EGFR) by acting as a competitive endogenous RNA (ceRNA) for miR-491-5p. Thus, circ_CELF1 could foster NSCLC development. Moreover, enforced expression of circ_CELF1 diminished the anticancer effects of anti-PD-1 therapy and shortened the survival time of NSCLC-bearing mice. Circ_CELF1 may represent a potential manageable target to overcome therapy resistance in NSCLC. Intensive research efforts should be encouraged to validate and expand this assumption.

The expression level of circHMGB2 was remarkably higher in NSCLC tissues than matched normal tissues (97). High expression of circHMGB2 was associated with poor prognosis of patients with LUAD and lung squamous cell carcinoma (LUSC). CircHMGB2 could upregulate coactivator-associated arginine methyltransferase 1 (CARM1) by sponging miR-181a-5p, thereby blocking the IFN-I response in LUAD and LUSC. These events contributed to formation of an immunosuppressive microenvironment and promotion of tumor growth in NSCLC-bearing mice. As expected, circHMGB2 overexpression profoundly reduced the therapeutic effect of PD-1 blockade *in vivo*. Pharmacological or genetic inhibition of CARM1 increased the sensitivity of circHMGB2-overexpressing NSCLC cells to anti-PD-1 treatment in murine cancer models. The interaction network between cancer cells and tumor microenvironment is complicated, and elucidating the underlying mechanisms is critically essential to preventing cancer immune evasion. Solid experimental data are still required to establish the role of circHMGB2 in the communication between cancer cells and tumor microenvironment.

## 4 Noncoding RNA-based anticancer immunotherapy

Due to the interconnection between ncRNAs and immunotherapy resistance, they are considered as prospective therapeutic targets for cancer intervention. Various mimics/antagonists of immunoregulatory miRNAs like miR-26, miR-33a, miR-34, miR-101, miR-125, miR-21, miR-31, miR-32, miR-100, miR-192 and miR-211 are under preclinical and clinical trials against cancer (105–108). However, none has yet reached the therapeutic breakthrough. The physiochemical characteristics of miRNA mimics/antagonists, such as susceptibility to nuclease-mediated degradation, off-target adverse effects and low cellular uptake, hinder their application in cancer treatment (109). Some strategies have been explored to solve the challenges associated with miRNA-targeted therapies. Chemical modifications such as locked nucleic acid (LNA), phosphorothioate-containing oligonucleotides and peptide nucleic acids (PNAs) could heighten the stability, cell targeting and uptake, and delivery efficacy of miRNA-based drugs (110). The engineering of targeted delivery formulations (e.g., liposomal- and polymeric-based delivery platforms) provides an extraordinary opportunity for the application of ncRNA-based therapeutics (111). Further clinical verifications are needed to position immunoregulatory ncRNAs as prospective therapeutic targets for better cancer management.

**Figure 7:**
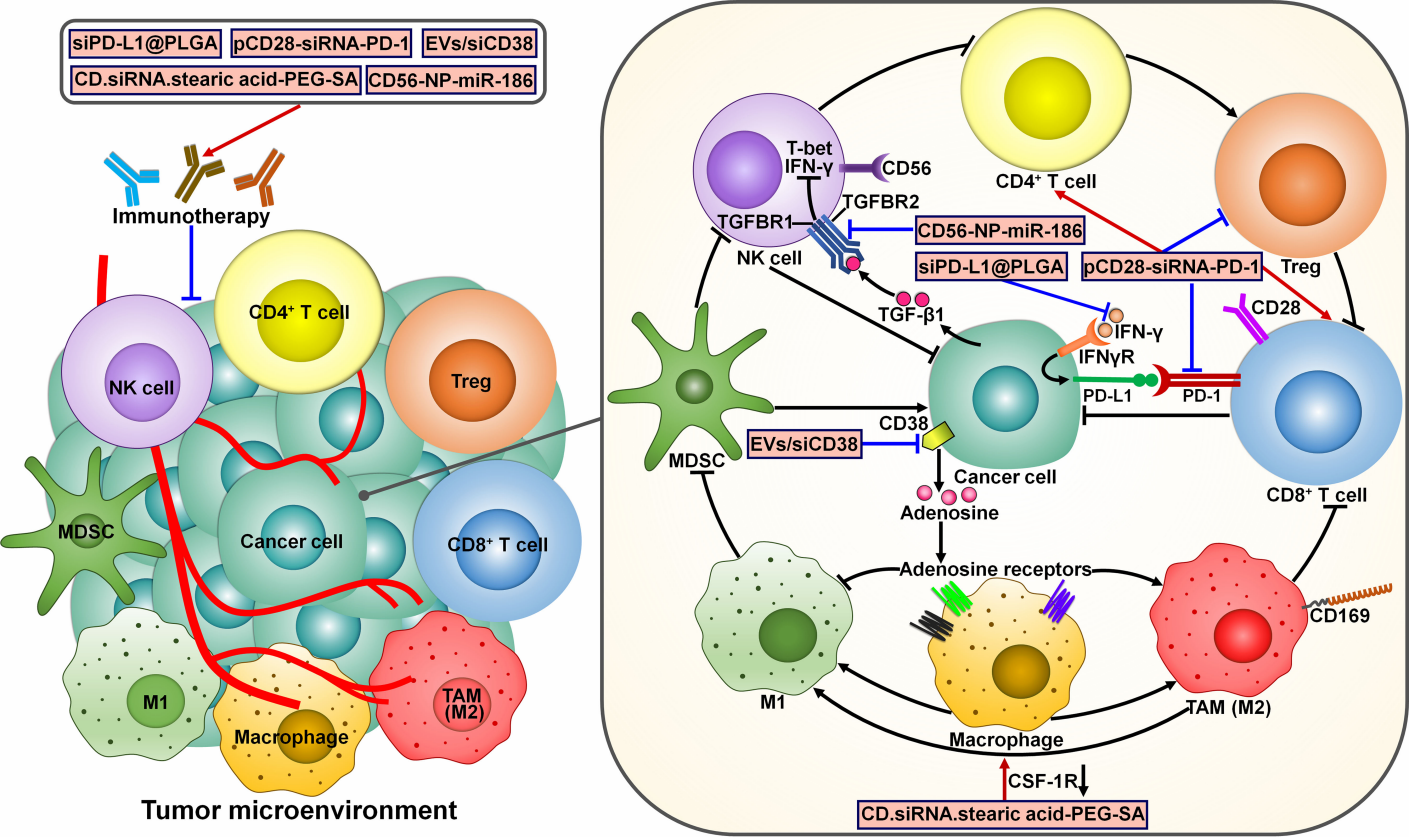
Mechanisms of action of potential ncRNA-based immunotherapeutics. Several ncRNA-centered immunotherapies have been developed. The TGF-β1/SMAD3 signaling pathway can inhibit the expression of T-bet and IFN-γ, thereby blunting NK cell activity. CD56-NP-miR-186 improves NK cytotoxicity by targeting TGFBR1 and TGFBR2. siPD-L1@PLGA suppresses IFN-γ-mediated PD-L1 induction and induces local expansion of CD8^+^ T cells. pCD28-siRNA-PD-1 can increase CD28 expression and reduce PD-1 expression. It also induces antitumor immune responses by increasing the populations of CD4^+^ T cells and CD8^+^ T cells while repressing Treg expansion. EVs/siCD38 impedes CD38-mediated adenosine generation and facilitates macrophage repolarization toward tumor-suppressive M1-like phenotype. CD.siRNA.stearic acid-PEG-SA induces the reprograming of M2 macrophages to M1 phenotype by silencing CSF-1R. These ncRNA-based approaches can reverse cancer resistance to conventional immunotherapies and hold great promise for the clinical use in cancer treatment. IFN-γ, interferon-γ; TGFBR1, transforming growth factor-β receptor 1; TGFBR2, transforming growth factor-β receptor 2; NK cell, natural killer cell; Treg, regulatory T cell; TGF-β1, transforming growth factor-β1; IFNγR, interferon-γ receptor; PD-L1, programmed cell death-ligand 1; PD-1, programmed cell death protein-1; MDSC, myeloid-derived suppressive cell; TAM, tumor-associated macrophage; CSF-1R, colony stimulating factor-1 receptor.

Several ncRNA-based immunotherapies have been proposed (**Figure 7**). For instance, a poly(lactic-co-glycolic acid) (PLGA)-based siRNA nanoparticle targeting PD-L1 (siPD-L1@PLGA) was previously generated and could be effectively captured by PDAC cells (112). The siPD-L1@PLGA impeded IFN-γ-mediated PD-L1 induction in recipient cells. Blockade of the PD-1/PD-L1 interaction via siPD-L1@PLGA induced local expansion of tumor-specific CTLs and strengthened the cytotoxicity of CTLs. Accordingly, the siPD-L1@PLGA increased the population of IFN-γ positive CD8^+^ T cells and markedly reduced tumor growth in a patient-derived xenograft (PDX) mouse model of pancreatic cancer. The upregulation of GzmB in siPD-L1@PLGA-treated pancreatic tumors implied high activity of NK cells or cytotoxic T cells. Given its low toxicity and facile production, the siPD-L1@PLGA could be a promising therapeutic option for PDAC immunotherapy. The extensive evidence outlined in this study warrants the pursuit of future studies to investigate the anticancer effects of this agent in PDAC patients. The co-expression plasmid of CD28-siRNA-PD-1 (pCD28-siRNA-PD-1) delivered by attenuated *Salmonella* dramatically prolonged the survival of melanoma-bearing mice (113). pCD28-siRNA-PD-1 elevated CD28 expression and suppressed PD-1 expression in tumor tissues. It also promoted cancer cell apoptosis by upregulating cleaved-caspase-3. Mice treated with pCD28-siRNA-PD-1 showed heightened antitumor immune responses, as evidenced by the increased number of NK cells, CD4^+^ T cells and CD8^+^ T cells and the decreased number of Tregs. These observations pave the way for developing novel combinatorial therapies that would enhance patient response to anti-PD-1 therapy. CD38 was found to be highly expressed in HCC (114). Extracellular vesicle (EV)-mediated transfer of CD38 siRNA (EVs/siCD38) facilitated macrophage repolarization toward M1-like phenotype and phagocytosis of cancer cells by macrophages through inhibition of CD38-mediated adenosine generation (114). Consequently, EVs/siCD38 exerted an inhibitory effect on HCC growth *in vitro* and *in vivo*. EVs/siCD38 also enhanced the sensibility of HCC cells to anti-PD-1/PD-L1 treatment in immunotherapy-resistant mice by attenuating the CD38 level. EVs/siCD38 may hold great promise for the further clinical application in cancer management. It is intriguing whether CD38 can directly modulate the function of immune cells in HCC, such as CD8^+^ T cells and Tregs. Special attention should be paid to the underlying mechanisms responsible for the anticancer efficacy of EVs/siCD38. A sialic acid-targeted cyclodextrin-based nanoparticle (CD.siRNA.stearic acid-PEG-SA) was previously developed to transport siRNAs to silence colony stimulating factor-1 receptor (CSF-1R) (115). Due to the interaction between sialic acid and its receptor Siglec-1 (CD169), the nanoparticle could specifically transfer CSF-1R siRNAs to M2 macrophages. The targeted formulation exhibited high levels of selective incorporation into M2 macrophages. As expected, the expression level of CSF-1R was profoundly decreased in M2 macrophages. CSF-1R downregulation induced the reprograming of M2 macrophages to M1 phenotype, leading to the apoptosis of prostate cancer cells. Altogether, the targeted cyclodextrin-based siRNA delivery system has potential to be a new immunotherapeutic measure for the intervention of prostate cancer. The tumor suppressor miR-186 was found to be present in NK cell-derived exosomes (116). miR-186 was downregulated in high-risk neuroblastoma patients, and its low expression was connected with poor prognosis of cancer patients. Anti-CD56 coated nanoparticles (CD56-NP) could specifically transfer the mature miR-186 to NK cells. CD56-NP-miR-186 blocked the transforming growth factor-β1 (TGF-β1)-dependent inhibition of NK cytotoxicity in high-risk neuroblastoma by targeting TGF-β receptor 1 (TGFBR1) and TGFBR2. Altogether, miR-186 counteracted TGF-β-dependent immune escape mechanism that hindered the efficiency of antibody-dependent cellular cytotoxicity (ADCC)-based therapies. Modulation of miR-186 abundance may be a feasible strategy to overcome immune evasion in neuroblastoma.

## 5 Prognostic and predictive value of long noncoding RNAs for immunotherapy in cancer

LncRNAs have been closely associated with both innate and adaptive immunity in cancer. Accordingly, immune-related lncRNAs have a significant impact on response to cancer immunotherapy. Several clinical trials have suggested the correlation between lncRNAs and clinical outcomes of cancer immunotherapy. A phase 2, single arm clinical trial showed that high expression of NF-κB-interacting lncRNA, *NKILA*, in cancer cells or cancer-specific CTLs predicted a poor prognosis among cancer patients treated with immunotherapy (117). A risk signature comprising seven ferroptosis-related lncRNAs (DUXAP8, LINC00894, LINC01426, LINC02609, MYG1-AS1, PELATON and PVT1) could predict the prognosis of patients with clear cell RCC (ccRCC) (118). Based on this prediction model, ccRCC patients were divided into low- and high-risk groups. Importantly, patients in low-risk group had better immunotherapy response than patients in high-risk group. A prognostic signature was previously constructed based on nineteen pyroptosis-related lncRNAs (e.g., LINC01704, LINC02345 and LINC02560) (119). This signature categorized HNSCC patients into low- and high-risk cohorts. The low-risk cohort was more sensitive to immunotherapeutic agents. A prognostic model consisting of eight lncRNAs AC004080.1, AC078923.1, AC114730.3, AC156455.1, LINC00997, LINC01419, NKILA and U62317.4 was developed (120). Patients with colon cancer were divided into low- and high-risk groups according to the prognostic model. Patients in low-risk group would benefit from immunotherapy. According to a risk signature comprising eight lncRNAs (AC011468.3, AC012306.2, AL441992.1, AP001453.2, AP001922.5, FZD4-DT, RUSC1-AS1 and SOX21-AS1), patients with cervical cancer were classified into different risk groups (121). High-risk patients were less sensitive to ICIs. LncRNA-based risk signatures have great significance to predict immunotherapy response and may aid in clinical decision-making for personalized treatment, but their clinical utility should be further validated.

## 6 Conclusions and future perspectives

T cell-targeted immunomodulators have been developed and approved for the treatment of various cancer types with an unprecedented speed over the past few decade. Although a tremendous step forward has been achieved, immunotherapy has not overcome existing hurdles of cancer treatment. Remarkably, emergence of resistance is a key factor restricting the efficacy of immunotherapy. NcRNAs, including miRNAs, lncRNAs and circRNAs, have the ability to alter the expression of genes involved in immune activation and suppression, and they also mediate the crosstalk between cancer cells and tumor immune microenvironment. It is not surprising that ncRNAs function as master regulators of immunotherapy resistance. Recent studies have revealed that ncRNAs are involved in cancer cell antigen presentation, immune escape, T cell priming and activation, reshaping of an immunosuppressive tumor microenvironment, cell death pathways during the immunotherapy of cancer. Continual studies are required to discover more ncRNAs that are involved in immune regulation and resistance. Comparison of the expression pattern of ncRNAs between immunotherapy-sensitive and immunotherapy-resistant cancer patient cohorts could be helpful in screening immune-related ncRNAs. The exact mechanisms through which ncRNAs affect immunotherapy response must be systematically defined. NcRNAs can affect both innate and adaptive immune responses. It is important to thoroughly explore the perplexing roles of ncRNAs in tumor immunity. LncRNAs and circRNAs are known to act as miRNA sponges and competitively bind to miRNAs, interfering with their effect on target gene expression. NcRNAs can act alone or crosstalk with each other to remodel the tumor immune microenvironment through different regulatory patterns. The overall impact of complex ncRNA interactions on immunotherapy response is worthy of further study. Notably, *in vitro* cell-based models or *in vivo* animal models cannot completely simulate the complicated state of the tumor microenvironment in cancer patients. Thus, the conclusions derived from preclinical studies must be interpreted with caution. Metabolic dysregulation in cancer cells can inhibit the intratumoral infiltration of immune cells and curtail antitumor immunity through the synthesis of immunosuppressive metabolites (122). Thus, aberrant cancer metabolism constitutes a critical mechanism of immunotherapy resistance. The causal link between ncRNA-mediated metabolic alteration in the tumor microenvironment and immunotherapy resistance has yet to be established, sparking continual research efforts in verifying this association. The composition and functionality of the tumor immune microenvironment differ across cancer types. Tumor immune microenvironment predominantly consists of DCs, lymphocytes (e.g., B cells, CD4^+^ T cells and CD8^+^ T cells), myeloid cells (e.g., macrophages, MDSCs and neutrophils) and NK cells (123). Tumor immune microenvironment participates in regulating cancer immune evasion and response to immunotherapy. Therefore, more studies investigating the influence of ncRNAs on the functionality of tumor immune microenvironment are required. The effects of ncRNAs on tumor immune microenvironment may be cancer type-specific. It will be important to adequately characterize the roles of ncRNAs in immune regulation across different cancer types. NcRNAs that are related to immunotherapy responses in various types of cancer may have potential to be exploited as future drug targets for effective cancer intervention.

So far, intensive efforts have been deployed to evaluate the therapeutic potential of ncRNA-centered treatments. Nevertheless, most results are still at the stage of basic or preclinical research. The success of immunotherapy mainly relies on cancer eradication through efficient activation of host immune system. Routine preclinical testing of anticancer agents in cancer cell lines or immune-compromised animals does not adequately take into account the engagement of the immune system. More reliable immune-competent preclinical models are key to understanding the anticancer action and mechanism of immunotherapies. Since ncRNAs play a vital role in various cellular processes under both physiological and pathological conditions, ncRNA-directed therapies, which are based on the functional repression of oncogenic ncRNAs through the application of antisense oligonucleotides and the replacement of tumor-suppressive ncRNA via the introduction of ncRNA mimics, may bring about significant detrimental outcomes. ICI immunotherapies work by reversing the physiological brakes of immune activation, so they may exert off-target effects leading to immune-induced inflammation of multiple organs. Accordingly, more extensive studies are warranted to completely evaluate the anticancer benefits and safe profiles of ncRNA-targeted therapies. Additional research efforts should be directed towards the optimization of immunotherapy regimens, with the aim to ameliorate the immunological-related adverse effects. Given the heterogeneity and complexity of immune states among cancer patients, it is essential to individualize ncRNA-based immunotherapy based on both cancer- and patient-specific characteristics. NcRNA-based immunotherapy could act in synergy with other treatment modalities including chemotherapy, radiotherapy and metabolic checkpoint-targeting therapies. The personalized combinatorial strategy may be further improved by determining the optimal sequencing of different treatments. Clinical translation of ncRNA-based therapeutics can be affected by delivery routes, immunogenicity and specificity. Many delivery vehicles have been utilized to transfer therapeutic ncRNAs, including lipid nanoparticles, metal-based nanoparticles, polymer-based delivery system and viral vectors (124). Exosomes and bacteriophages are considered as ideal and favorable transporters of ncRNA therapeutics in clinical practice due to their poor immunogenicity and high delivery efficiency. NcRNA oligonucleotides are mainly recognized by Toll-like receptors (TLRs), leading to adverse immune reactions. Chemical modification is regarded as a potential approach to reduce the immunogenicity of ncRNA-based therapeutics. However, even after chemical modification in ncRNA oligonucleotides, this issue is not well addressed yet and should be further explored. Increasing knowledge of TLR-associated adverse immune effects will assist in developing novel ncRNA therapies that exhibit low immunogenicity before the initiation of clinical trials. To reduce the detrimental effects of ncRNA mimics or antagonists in undesired cells, expression vectors carrying promoter sequences whose expression is specifically upregulated in target cells could be utilized to express therapeutic ncRNA oligonucleotides. The specificity of ncRNA-based therapeutics may be further improved by targeting the precursors of ncRNAs to inhibit their synthesis and maturation using nucleic acid oligonucleotides or peptides. Despite encouraging preclinical and early clinical evidence, the successful clinical utility of ncRNA-based immunotherapy has not yet been realized. With increasing knowledge of ncRNA involvement in tumor immunity and immunotherapy, there is hope for ncRNA-based therapeutics as novel treatments for cancer.

## Author contributions

MW: Conceptualization, Funding acquisition, Investigation, Supervision, Visualization, Writing – original draft. FY: Resources, Writing – review and editing, Visualization. PL: Project administration, Supervision, Writing – review and editing.
